# IMARI: multi-Interventional program for prevention and early Management of Anastomotic leakage after low anterior resection in Rectal cancer patIents: rationale and study protocol

**DOI:** 10.1186/s12893-020-00890-w

**Published:** 2020-10-15

**Authors:** M. D. Slooter, K. Talboom, S. Sharabiany, C. P. M. van Helsdingen, S. van Dieren, C. Y. Ponsioen, C. Y. Nio, E. C. J. Consten, J. H. Wijsman, M. A. Boermeester, J. P. M. Derikx, G. D. Musters, W. A. Bemelman, P. J. Tanis, R. Hompes, on behalf of the IMARI-study group, J. D. W. van der Bilt, J. W. A. Burger, R. M. P. H. Crolla, F. Daams, I. Faneyte, M. Gerhards, E. J. R. de Graaf, W. J. de Jonge, W. van der Meij, S. J. Oosterling, L. P. S. Stassen, J. B. Tuynman, E. G. G. Verdaasdonk, H. L. van Westreenen, J. H. W. de Wilt

**Affiliations:** 1 Department of Surgery, Amsterdam UMC, Location AMC, Amsterdam, The Netherlands; 2 Department of Gastroenterology, Amsterdam UMC, Location AMC, Amsterdam, The Netherlands; 3 Department of Radiology, Amsterdam UMC, Location AMC, Amsterdam, The Netherlands; 4 Department of Surgery, Meander Medical Center, Amersfoort, The Netherlands; 5 Department of Surgery, Amphia Hospital, Breda, The Netherlands; 6 Department of Paediatric Surgery, Emma Children’s Hospital, Amsterdam UMC, Location AMC, Amsterdam, The Netherlands

**Keywords:** Rectal cancer, Anastomotic leakage, Total Mesorectal excision, Prevention, Anastomotic salvage

## Abstract

**Background:**

Anastomotic leakage (AL) is still a common and feared complication after low anterior resection (LAR) for rectal cancer. The multifactorial pathophysiology of AL and lack of standardised treatment options requires a multi-modal approach to improve long-term anastomotic integrity. The objective of the IMARI-trial is to determine whether the one-year anastomotic integrity rate in patients undergoing LAR for rectal cancer can be improved using a multi-interventional program.

**Methods:**

IMARI is a multicentre prospective clinical effectiveness trial, whereby current local practice (control cohort) will be evaluated, and subsequently compared to results after implementation of the multi-interventional program (intervention cohort). Patients undergoing LAR for rectal cancer will be included. The multi-interventional program includes three preventive interventions (mechanical bowel preparation with oral antibiotics, tailored full splenic flexure mobilization and intraoperative fluorescence angiography using indocyanine green) combined with a standardised pathway for early detection and active management of AL. The primary outcome is anastomotic integrity, confirmed by CT-scan at one year postoperatively. Secondary outcomes include incidence of AL, protocol compliance and association with AL, temporary and permanent stoma rate, reintervention rate, quality of life and functional outcome. Microbiome analysis will be conducted to investigate the role of the rectal microbiome in AL.

In a Dutch nationwide study, the AL rate was 20%, with anastomotic integrity of 90% after one year. Based on an expected reduction of AL due to the preventive approaches of 50%, and increase of anastomotic integrity by a standardised pathway for early detection and active management of AL, we hypothesised that the anastomotic integrity rate will increase from 90 to 97% at one year. An improvement of 7% in anastomotic integrity at one year was considered clinically relevant. A total number of 488 patients (244 per cohort) are needed to detect this difference, with 80% statistical power.

**Discussion:**

The IMARI-trial is designed to evaluate whether a multi-interventional program can improve long-term anastomotic integrity after rectal cancer surgery. The uniqueness of IMARI lies in the multi-modal design that addresses the multifactorial pathophysiology for prevention, and a standardised pathway for early detection and active treatment of AL.

**Trial registration:**

Trialregister.nl (NL8261), January 2020.

## Background

Anastomotic leakage (AL) is still a common and feared complication after low anterior resection (LAR) for rectal cancer. A nationwide cross-sectional study with more than 3-years follow-up revealed an overall incidence of 20% [1]. Occurrence of AL leads to significant increase of postoperative morbidity, prolonged hospital stay, increased healthcare costs, and adversely affects oncological and functional outcome with an increased risk of a permanent stoma [2–4]. The underlying aetiology for AL is a complex multifactorial mix of both modifiable and non-modifiable risk factors that relate to various patient- and tumour characteristics, neo-adjuvant protocols and intraoperative technical aspects [1, 5–7]. Examples of modifiable surgical factors include tension on the anastomosis and anastomotic perfusion. Lately, the impact of the gut microbiome on AL has been studied and a pivotal role seems plausible [8, 9].

While better understanding and modification of risk factors will undoubtedly drive AL rates down, the risk will never be completely non-existent as a result of non-modifiable and currently unknown factors. Hence, besides focus on prevention, limiting the impact of AL is equally important and can be achieved by early detection and appropriate management. However, no international consensus exists on a diagnostic pathway for early detection of AL, even though evidence is building for the use of C-reactive protein (CRP) in the early postoperative period [10, 11]. Regarding management of AL, this usually involves a deviating ileostomy if not yet performed primarily, in combination with “passive” drainage of the abscess cavity via transanal or percutaneous route [1, 12]. Using this aforementioned approach, almost half of the leaks do not heal and may require major salvage surgery, including the creation of a permanent stoma [1, 12].

We hypothesised that a multi-interventional program with a focus on prevention, diagnosis and management of AL would improve the one-year anastomotic integrity rate in patients undergoing LAR for rectal cancer. In the IMARI trial, the chosen set of interventions aiming at reduced risk of AL were: (1) mechanical bowel preparation (MBP) with oral antibiotics (AB) to optimise the microbiome [13–16]; (2) splenic flexure mobilization to optimise a tension-free anastomosis [17]; (3) intraoperative real-time fluorescence angiography (FA) using indocyanine green (ICG) to assess adequate perfusion [18, 19]. These preventive measures are combined with clinical pathways for early detection and “active” management of AL. Serial CRP measurements in the early postoperative period in combination with a CT-scan with rectal contrast will be employed for early detection. On confirmation of AL, endoscopic vacuum-assisted closure therapy (EVAC) of the abscess cavity is initiated to control pelvic sepsis followed by early transanal closure or restorative re-do surgery to restore anastomotic integrity. This quality controlled multi-interventional program will be implemented within existing institutional enhanced recovery programs and prehabilitation initiatives.

## Methods

This study protocol is written in accordance with the SPIRIT guidelines [20, 21] and the SPIRIT checklist is provided in Appendix 1.

### Study objectives

The primary objective of this study is to determine whether the one-year anastomotic integrity rate in patients undergoing LAR for rectal cancer can be improved using a multi-interventional program which includes: (1) MBP/AB; (2) tailored full splenic flexure mobilisation; (3) intraoperative FA using ICG ; (4) routine CRP measurements postoperatively and CT-scan with rectal contrast on indication; (5) EVAC with early transanal closure of the anastomotic defect or restorative re-do surgery.

Secondary objectives include the evaluation of the multi-interventional program on the AL rate and quality of life until one year after the index operation, and the establishment of the IMARI biobank. The rationale for sample collection in the IMARI biobank is to investigate the role of the rectal microbiome in AL.

### Study design

**Fig. 1:**
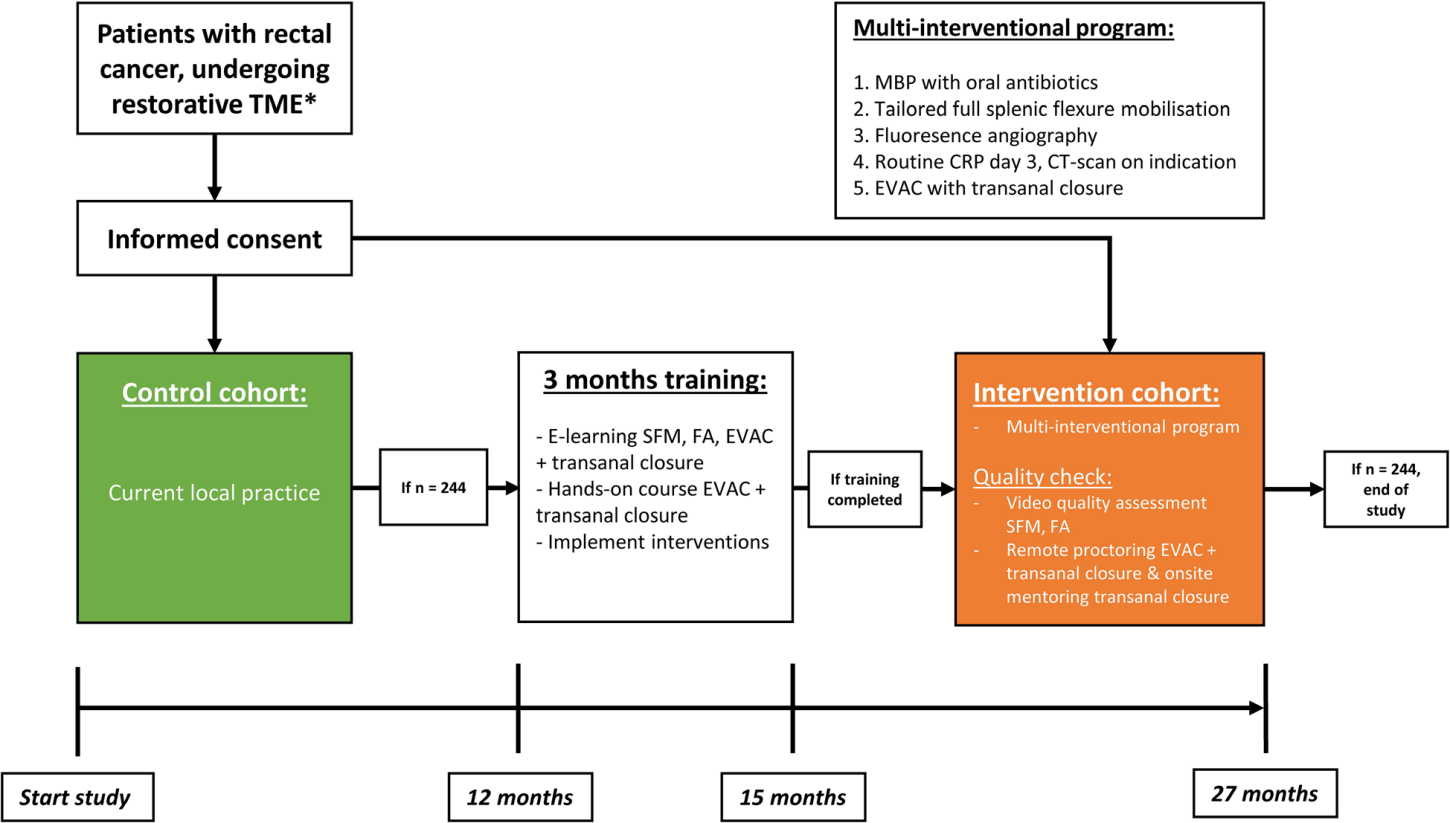
Flow diagram study. MBP, Mechanical Bowel preparation; CRP, C-reactive protein; CT, computed tomography; EVAC, endoscopic vacuum-assisted closure; FA, Fluorescence angiography; SFM, Splenic flexure mobilisation; TME, Total Mesorectal Excision

The IMARI trial is a multicentre prospective clinical effectiveness trial, whereby current local practice (control cohort) will be evaluated, and subsequently compared to results after implementation of the multi-interventional program (intervention cohort). The flow diagram for the study is shown in Fig. 1.

### Ethical consideration

The trial will be conducted according to Good Clinical Practice guidelines and the principles of the declaration of Helsinki (2013, [22]). This study is approved by the Medical Ethical Committee and Biobank committee of the Amsterdam UMC, location AMC. The protocol is registered by the Dutch Central Committee on Research Involving Human Subjects (NL67600.018.18) and is submitted to the trialregister.nl database (NL8261).

### Study population

Eligibility criteria for study participation are: (1) planned to undergo LAR for either one of the following diagnoses: a) primary rectal cancer as defined by the international consensus definition for rectal cancer [23] or b) regrowth of rectal cancer in a watch and wait protocol or c) completion/salvage surgery after local excision for rectal cancer; (2) willing to complete quality of life questionnaires and comply with schedule of outpatient follow-up visits; (3) ≥ 18 years old.

A subject is not eligible for inclusion in case of presence of one of the following exclusion criteria: (1) LAR without colorectal or coloanal anastomosis; (2) locally advanced rectal cancer, expected to require beyond-total mesorectal excision approach or multi-visceral excision; (3) synchronous colonic resections.

### Informed consent procedure

Patients meeting all eligibility criteria stated above will be informed on the trial at the outpatient clinic by a member of the research team. Written informed consent will be obtained for participation in the trial and separate consent obtained for storage of samples in the IMARI biobank. Every included patient will be assigned a three-digit study number and only local sites have access to a decryption code.

### Study outline

#### Control cohort

The study will start in all participating hospitals with accrual into the control cohort, whereby patients will receive care according to standard local protocol. The local protocol may well include one or more components of the multi-interventional program and this will be recorded in the case-report form (CRF) for each patient.

#### Intervention cohort

When accrual of the control cohort has been completed (*n* = 244, Fig. 1), all participating hospitals will start a training period of 3 months before implementation of the multi-interventional program and accrual of patients into the intervention cohort. A standardised protocol for MBP/AB and postoperative surveillance of patients for AL will be distributed among centres, enabling timely implementation before start of the intervention cohort. Staff from participating centres will be trained via online educational modules and hands-on training sessions on tailored splenic flexure mobilization, intraoperative FA and EVAC management of AL combined with early surgical closure of anastomotic defects. Random checks of procedural videos and use of a system for remote proctoring will be employed to ensure quality control throughout the entire trial period.

#### Multi-interventional program

##### Mechanical bowel preparation with oral antibiotics

MBP will start the day before surgery by oral administration of 2 l of polyethylene glycol (Moviprep®) or sodium phosphate. Based on the results from the SELECT-trial [16] and unpublished work from the pre-caution trial [24], 10 ml of selective digestive decontamination (SDD) solution will be administered four times daily during the three days prior to surgery. The SDD suspension (10 ml) will contain: colistine 100 mg, tobramycine 80 mg and amphotericine B 500 mg.

##### Tailored full splenic flexure mobilization

For low rectal cancers, defined according to the LOREC definition, a full splenic flexure mobilisation is mandatory [25, 26]. For all other rectal cancers a full splenic flexure mobilisation is at the discretion of the operating surgeon. Full splenic flexure mobilisation entails the following essential and mandatory steps: (1) division of the inferior mesenteric vein at the lower border of the pancreas just lateral to the angle of Treitz; (2) full release of the distal transverse colonic mesentery from the body and tail of the pancreas; (3) division of the gastro-colic ligament to release omentum from distal transverse colon. These steps can be completed either in a medial to lateral or lateral to medial approach.

##### Intraoperative fluorescence angiography using indocyanine green

Intraoperative FA using ICG will be performed in all patients before and after construction of the anastomosis using a standard intravenous injection of ICG (0.1 mg/kg/bolus). Near infrared imaging can be performed by different imaging platforms, and all relevant FA characteristics will be recorded in the CRF. The first assessment is done after rectal mobilisation, but prior to bowel division. The proximal colon will be assessed under conventional white light and the point of planned transection will be marked. Subsequently, FA will be performed using either an intracorporeal or extracorporeal FA technique. The decision whether or not to change the planned anastomotic site will be made according to the surgeon’s subjective interpretation of FA.

Anastomotic reconstruction is performed according to the surgeon’s preference, followed by an intracorporeal or intraluminal FA assessment of the anastomosis after a second bolus of ICG. Any anastomotic revision, or additional manipulation of the anastomosis (i.e. sutures) will be recorded. The creation of a deviating stoma will be at the surgeon’s discretion. A third dose of ICG is allowed, if deemed necessary by the operating surgeon.

##### Routine CRP measurement

**Fig. 2:**
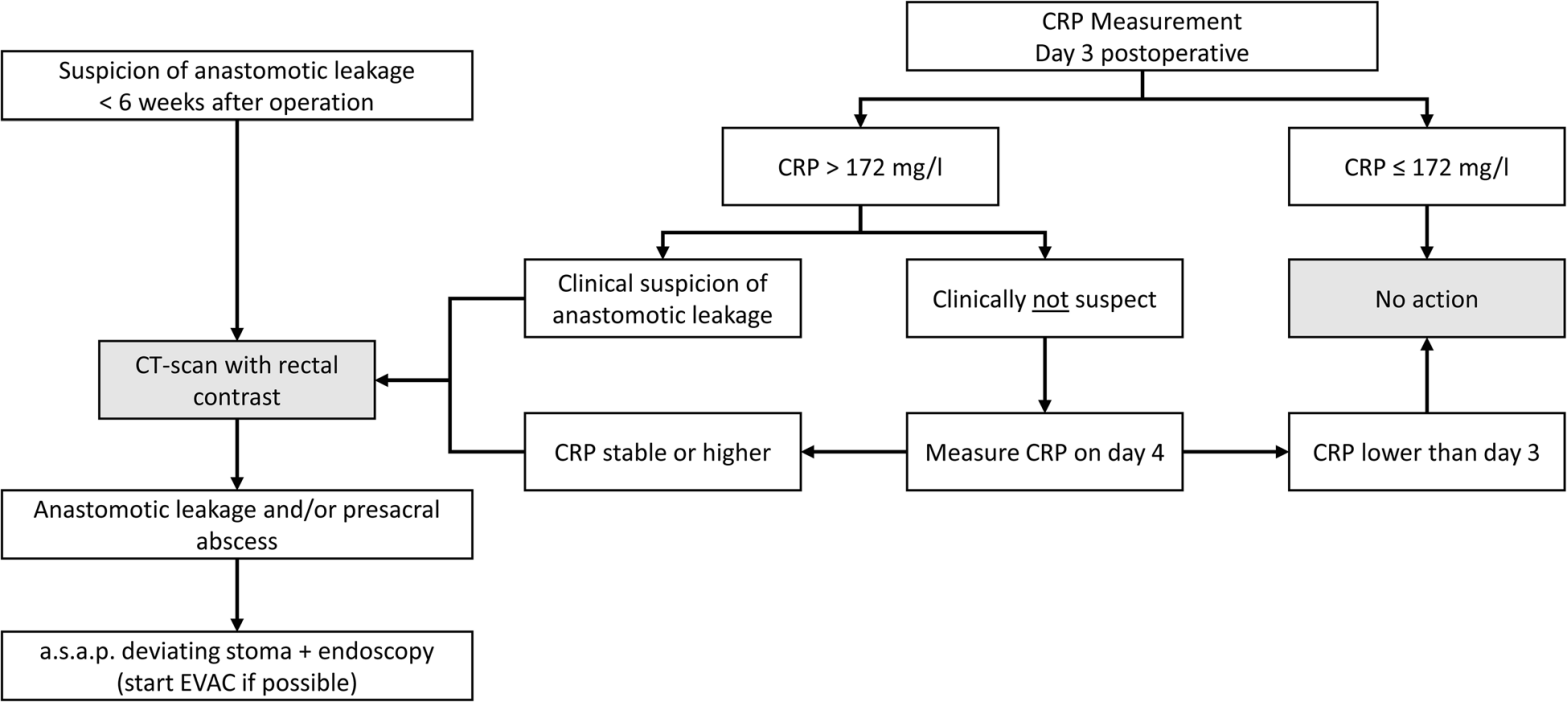
Flow diagram postoperative algorithm

CRP measurement will be performed routinely on day 3 postoperatively. A CRP level above the threshold of 172 mg/l [10], combined with any clinical aberrant observations, will trigger a CT Abdomen with rectal contrast. Otherwise, CRP measurement will be repeated at day 4 postoperatively. In case of a stable or higher CRP level, a CT abdomen with rectal contrast will be performed to exclude AL, irrespective of clinical findings. Any extraluminal air and/or fluid at the level of the anastomosis will at least be considered as suspicious of AL based on CT, requiring further investigation. Any extravasation of contrast will be defined as clear AL. The algorithm for clinical decision making according to CRP level is displayed in Fig. 2.

##### Endoscopic vacuum-assisted drainage with early transanal closure of the anastomotic defect

**Fig. 3:**
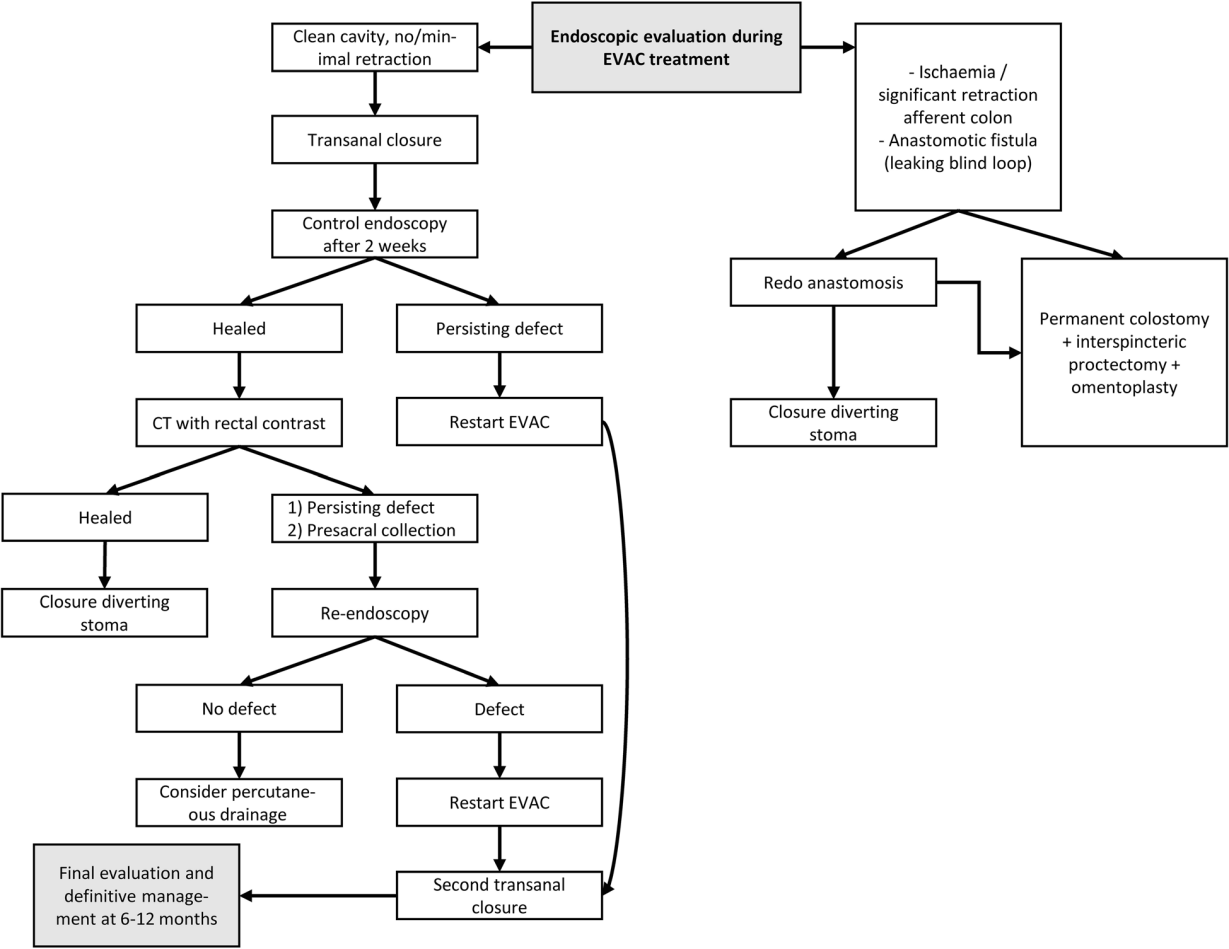
Flow diagram pro-active leak management

When the CT-scan reveals clear AL, clinical management depends on the presence of a primary diverting stoma. If not created primarily, a diverting ileostomy will be constructed with abdominal lavage in case of purulent or fecal peritonitis, preferably using a laparoscopic approach, and combined with intraoperative endoscopic assessment of the anastomosis with EVAC if indicated. In patients with primary diversion, endoscopic assessment of the anastomosis can be performed under general anaesthesia, especially if surgical management of peritonitis is required, or under sedation at the endoscopy room. For a pelvic fluid collection on CT without any obvious extraluminal contrast, an endoscopy is preferred as first step to assess whether an actual defect can be identified before return to theatre for diversion. At endoscopy, potential signs of ischaemia and characteristics of the anastomotic defect (extent circular dehiscence, retraction) will determine further steps to control pelvic sepsis (Fig. 3).

Patients deemed suitable for EVAC will have endosponge exchanges every 3–4 days, with assessment of the anastomotic defect and associated cavity by the gastroenterologist and/or surgeon. Usually after two to four endosponge exchanges, the anastomotic defect should be ready to be closed transanally as previously described [27–29]. The transanal closure will be checked by endoscopy two weeks postoperatively. If no defect is identified at endoscopy, a further assessment will follow by CT with rectal contrast. At the time of endoscopy a CRP check will also be included.

If the initial endoscopic evaluation reveals ischaemia or significant retraction of the afferent colon, a different pathway will be followed: (1) early or late re-do of the anastomosis, with use of EVAC for initial control of pelvic sepsis; or (2) take down of the anastomosis; preferred technique will be intersphincteric resection of the rectal remnant, permanent colostomy and filling of the pelvis with an omentoplasty.

At any point in time, participating centres can contact the initiating centre for advice, assessment of endoscopy images and the most appropriate further step in management of the AL and sepsis.

### Outcomes

The primary outcome of this study is anastomotic integrity one year after the index operation. This will be determined in all included patients by CT-scan at one year as part of regular follow-up of patients after rectal cancer surgery [30].

Secondary outcomes include: (1) incidence of AL within 30 days, 90 days, and one year post-operative; (2) protocol compliance to any intervention; (3) protocol compliance in association to AL; (4) changes in rectal microbiome and association with AL; (5) permanent stoma rate; (6) temporary stoma rate and total time of having a stoma during one year; (7) length of hospital stay after index surgery and total stay during one year; (8) overall and stoma-related readmission and reintervention rates; (9) quality of life (EQ-5D, QLQ-C30, QLQ-CR29, 10) bowel, urinary and sexual function (LARS, UDI-6, IIQ-7, IIEF for male and MFSFQ for female) pre-operatively, at 90 days and one year; (11) diagnostic accuracy of serial CRP at day 3–4; (12) efficacy of EVAC with early transanal closure of the anastomotic defect; (13) change of management related to FA: site of proximal bowel division used for anastomosis, re-do anastomosis, reinforcement of anastomosis after construction, decision for diverting stoma, or decision for a non-restorative procedure; (14) operative and post-operative complications within 90 days of index surgery; (15) 1-year local recurrence and overall survival rate.

To assess the rectal microbiome, the following samples are collected for the IMARI biobank: stool samples before start MBP/AB and at day 4 postoperative, the anastomotic donut (colonic side) from the operation, intraoperative rectal swab from the anastomotic site, and for patients that develop AL an endoscopic rectal swab from the abscess cavity. Samples will be stored centrally in the IMARI biobank at the Tytgat Institute in the Amsterdam UMC, location AMC. Microbiota profiling will be done using an Illumina Miseq platform. In addition, metatranscriptomics will be performed on selected samples to look for presence and activity of collagenolytic *Enterococcus faecalis* and additional detrimental species for anastomotic integrity.

Collection points of all outcomes are summarised in Table 1.

**Table 1:**
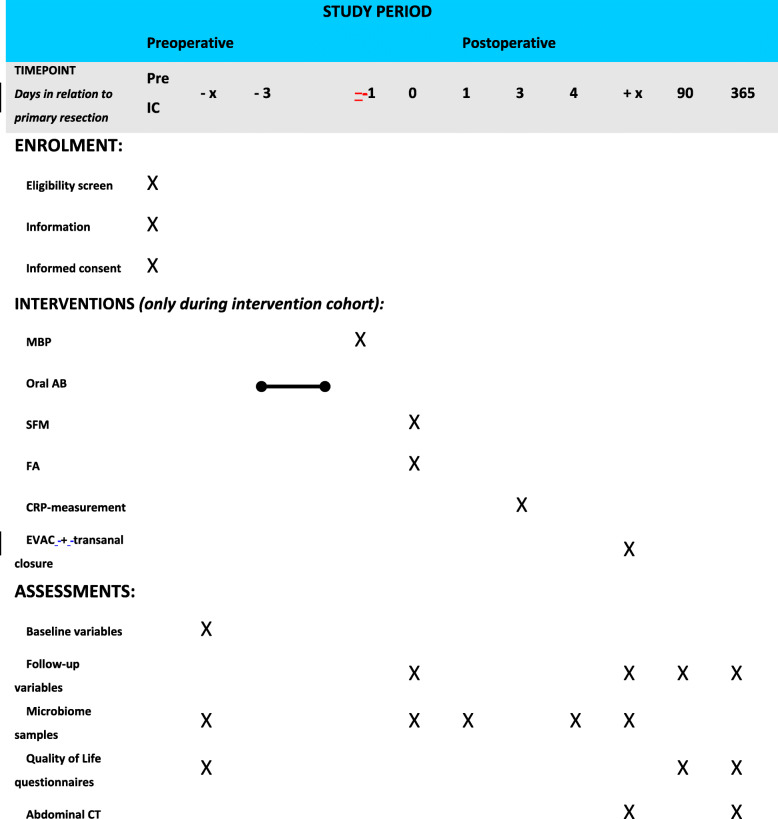
Timing of enrollment, interventions and assessments. IC, informed consent

### Sample size calculation

In a Dutch nationwide study, the AL rate was 20%, with anastomotic integrity of 90% after one year [1]. Meta-analysis of MBP/AB revealed that preoperative antibiotics were associated with lower AL rates (OR 0.59, 0.53–0.67; *p* < 0.001) [14]. Pooled analysis of studies using routine FA showed an OR of 0.34 (0.16–0.74; *p* = 0.006) [18]. Together with full splenic flexure mobilisation, the estimated reduction in AL rate is 50%. In the CLEAN-study, treatment with EVAC and early surgical closure resulted in anastomotic healing in two thirds of the patients within the first year [31]. Therefore, we hypothesised that the combination of all interventions will increase the anastomotic integrity rate from 90 to 97% at one year. Applying a Fisher exact test with a two-sided 0.05 significance level and 80% power, and with an estimated drop-out of 10%, a total number of 488 patients (244 per cohort) are needed to be able to detect a 7% increase in anastomotic integrity by implementation of the combined interventions.

### Statistical analysis

The primary endpoint, anastomotic integrity, will be compared between the two trial cohorts using a two-sided Fisher exact test. AL rates will be compared between the cohorts using generalised estimating equations model adjusting for stratification factors. This approach will be used to test the two-sided hypothesis that the AL rate is equal in both cohorts (i.e. an odds ratio of 1), considering the 95% confidence interval and a *p*-value of 0.05. Other secondary endpoints with binary measures will be analysed using multi-variable logistic regression adjusting for stratification factors. Secondary endpoints with continuous measures will be analysed using linear regression models adjusting for stratification factors. When the data is not normally distributed, the data will be transformed to achieve normal distribution. The secondary endpoint ‘duration of temporary stoma’ will be analysed using a cox-regression model with adjusting for stratification factors. Quality of life and function outcome will be calculated as domain and summarised scores according to the manuals, and graphically represented across all time points. Comparisons of questionnaire outcomes will be analysed using linear mixed models. Statistical analyses will be performed using the latest version of SPSS software for Windows.

The statistical analysis plan will be finalised before data is locked for analysis, and decision will be made on stratification factors and planned subgroup analysis, and on how to deal with application of components of the multi-interventional program in the control cohort, protocol violations, and baseline imbalance.

### Safety reporting

This IMARI trial is considered a low-risk study, because any of the interventions are already being used in routine daily practice. Serious adverse events will not be reported for the control cohort, since patients will receive standard care. Serious adverse events will be recorded until 30 days after index surgery or any study related procedure for the intervention cohort.

### Data handling and monitoring

Data will be digitally collected using the electronic data management system Castor EDC (www.castoredc.com). In all participating hospitals, one surgeon acts as local investigator who is primarily responsible for execution of trial interventions, and for accuracy and completeness of the CRF. Quality of life questionnaires will be collected through the data collection initiative of the Prospective Dutch ColoRectal Cancer (PLCRC) group (clinicaltrials.gov NCT02070146). This study will be monitored as described in a monitoring plan by an independent monitor to ensure quality and adherence to the protocol. If patients are only willing to participate in the IMARI-trial, questionnaires will be collected by the investigators.

### Public disclosure and publication policy

IMARI was registered at the trialregister.nl database (NL8261). The results of IMARI will be submitted to a peer-reviewed journal regardless of study outcome. Co-authorship will be based on the international ICMJE guidelines. Besides the key authors (coordinating investigators as first authors and principal investigators as senior authors), authorship is granted to the local investigator of each centre when at least ten patients are included in the trial and when substantial contribution to the trial is made.

## Discussion

In contrast to improvements over the last decades regarding oncological outcomes after rectal cancer surgery, AL and ensuing long-term sequelae remain common. A cross-sectional study in the Netherlands revealed an AL rate of 20% after long-term follow-up, with nearly half of AL not healing and giving rise to a chronic sinus. In the IMARI trial we propose a multi-interventional program, not only being designed to reduce AL, but also to increase the chance of long-term anastomotic integrity. The uniqueness of the IMARI trial lies in the multi-modal design that addresses the multifactorial pathophysiology, early detection and active treatment of AL.

Thus far, many risk factors have been associated with AL and a complex multifactorial pathophysiology has emerged [1, 5–7, 9]. Most interventional studies up till now only evaluated the impact of a single risk factor on AL [16, 17, 32, 33]. The IMARI trial addresses three modifiable risk factors to ensure a tension-free, adequate perfused anastomosis, under optimal condition of the microbiome: (1) MBP/AB that could lead to a reduction in AL by reduction of the fecal bulk and bacterial load [13–16]; (2) Splenic flexure mobilization to optimise a tension-free anastomosis, particularly for low rectal cancer [17, 34]; (3) Intraoperative real-time FA using ICG to assesses adequate perfusion of the afferent colon and anastomosis. Routine use of this FA technology has been associated with reduced AL rates, although no data from large randomised controlled trials (RCT) are available [18, 19].

If AL occurs, prompt detection is crucial to allow for immediate treatment initiation and control of pelvic sepsis. Rapid sepsis control avoids further morbidity and should also limit long-term functional sequelae. Although transanal and/or radiological transgluteal drainage of pelvic sepsis does allow for some degree of sepsis control, leakage is not actively treated and the anastomotic defect is not likely to heal spontaneously. In contrast, after 2–4 EVAC exchanges, which takes approximately 1–2 weeks, well vascularised granulation tissue is often visible inside the cavity. This allows for subsequent transanal closure of the anastomotic defect with a suction drain positioned behind the anastomosis with its tip inside the cavity, after which the cavity collapses and the neo-rectum expands [29, 31]. As such, EVAC in combination with early transanal closure allows for a more active, rapid control of pelvic sepsis and at the end mucosal approximation. This pathway should allow for more anastomoses to be preserved, prevent chronic presacral sinuses and improve functional outcomes by limiting peri-anastomotic fibrosis with preservation of compliance of the neo-rectum.

Even though RCTs are considered the most robust research strategy for establishing a causal relationship, a comparative cohort design was chosen for the IMARI trial. In the setting of a classical RCT, contamination is likely to occur in the control arm. Surgeons are likely to change their daily practice, when observing benefits from the multi-interventional program. We consider this also a problem in a stepped-wedge cluster RCT, a frequently used variant of a classical RCT. Thus, a comparative cohort design was selected in the form of a prospective clinical effectiveness trial, where crossover to the intervention cohort occurs after completion of accrual in the control cohort. Participating centres will simultaneous start recruitment for the intervention arm, after completion of a 3 month training period. Furthermore, in the set-up of a clinical effectiveness trial the true impact of utilising the multi-interventional program can be evaluated under real conditions [35].

For the purpose of the IMARI trial, a multidisciplinary scientific study-group was composed, including surgeons from both academic and peripheral centres, gastroenterologists, radiologists, specialised nurses and researchers. In this way hospital-wide awareness is created and a broadly supported multi-modal approach was made possible.

Successful implementation of the IMARI multi-interventional program within existing enhanced recovery and prehabilitation programs would have a positive influence on morbidity, mortality, and possibly oncological outcomes. By increasing the chance of long-term anastomotic integrity and decreasing permanent stoma rates, the IMARI trial should contribute to a better quality of life for patients undergoing rectal cancer surgery.

## Supplementary information

**Additional file 1.**

## Data Availability

Data collection is in progress. When data collection and follow-up is finalized, data from the study will be available on reasonable request from the corresponding author.

## Abbreviations

AB: Antibiotics
AL: Anastomotic Leakage
CRP: C-reactive protein
CT: Computed tomography
EDC: Electronic Data Capture
EVAC: Endoscopic vacuum-assisted closure
FA: Fluorescence angiography
ICG: Indocyanine green
ICMJE: International Committee of Medical Journal Editors
LAR: Low anterior resection
LOREC: Low Rectal Cancer Development programme
MBP: Mechanical bowel preparation
PLCRC: Prospective Dutch ColoRectal Cancer
RCT: Randomized controlled trial
SDD: Selective digestive decontamination
SPIRIT: Standard Protocol Items: Recommendations for Interventional Trials
TME: Total mesorectal excision
